# Myocardial fibrosis and ventricular strain indices in post-fontan single ventricle patients: cardiac MR assessment and prognostic significance

**DOI:** 10.1186/1532-429X-15-S1-O99

**Published:** 2013-01-30

**Authors:** Sanmit K Basu, Patrick T Broderick, Brian P Cupps, Michael K Pasque, Glenn J Foster, C Huie Lin, Pamela K Woodard, Charles E Canter, Gautam K Singh

**Affiliations:** 1 Department of Pediatrics, Division of Cardiology, St. Louis Children's Hospital, Saint Louis, MO, USA; 2 Department of Surgery, Division of Cardiothoracic Surgery, Washington University School of Medicine, Saint Louis, MO, USA; 3 Mallinckrodt Institute of Radiology, Washington University School of Medicine, Saint Louis, MO, USA; 4 Department of Medicine, Cardiovascular Division, Washington University School of Medicine, Saint Louis, MO, USA

## Background

Staged palliation culminating with the Fontan procedure has dramatically increased survival in children with single ventricle anatomy. However, long-term morbidity and mortality continue to be significant. This has been linked to ventricular myocardial fibrosis and scarring and subsequent altered contractile mechanics with abnormal regional wall motion and strain, but has not been fully elucidated. In this study, we sought to more precisely characterize these structural changes via magnetic resonance imaging and quantitatively assess ventricular mechanics through the measurement and calculation of MRI-derived displacement fields and regional wall strain to discern any association between fibrosis and function in the post-Fontan population.

## Methods

The study population consisted of patients with single systemic ventricle anatomy of left ventricular morphology. Inclusion criteria included age > 5 years, Fontan completion > 2 years from time of MR study, and ability to undergo MRI without sedation. Studies on 22 Fontan subjects and 39 normal control subjects were completed. The MR protocol included cine bright-blood sequences for function, SPAMM cine gradient-recalled echo sequences for tagged analysis, and delayed contrast enhancement with gadolinium for tissue characterization. The tagged images were used to calculate a 3-dimensional LV myocardial displacement field via a validated methodology incorporating interpolation of tag line and surface deformation and finite element approximation. Strain values throughout the myocardium were then calculated from this continuous displacement field. Delayed myocardial enhancement was evaluated using standard techniques to identify regions of fibrosis. All strain values were expressed as means with standard deviations, and two-way ANOVA was utilized to compare strain in normal and fibrotic regions.

## Results

In the Fontan cohort analyzed thus far, three distinct myocardial fibrosis/scarring patterns have been identified in about half the subjects - localized subendocardial, transmural, and diffuse. In these areas, altered contractile mechanics were present with decreased regional and global strain. Compared to normal subjects, in whom no fibrosis or scarring was demonstrated, circumferential and longitudinal strains were significantly (p < 0.05) decreased - circumferential: -0.18 +/- 0.07 (Fontan) vs. -0.26 +/- 0.04 (normal), longitudinal: -0.13 +/- 0.06 (Fontan) vs. -0.21 +/- 0.04 (normal). Furthermore, median systemic ventricular ejection fraction was also decreased in the Fontan cohort versus the normal cohort - 42% vs. 65%.

## Conclusions

In the post-Fontan population, fibrotic LV myocardial segments demonstrate decreased thickening and abnormal wall motion with decreased regional strain measures and global ventricular EF. Cardiac MR techniques are a valuable tool to assess myocardial characteristics and contractile mechanics in these patients.

## Funding

No current funding.

